# The Current Status and Development of Insect-Resistant Genetically Engineered Poplar in China

**DOI:** 10.3389/fpls.2018.01408

**Published:** 2018-09-21

**Authors:** Guiying Wang, Yan Dong, Xiaojie Liu, Guosheng Yao, Xiaoyue Yu, Minsheng Yang

**Affiliations:** ^1^Institute of Forest Biotechnology, Forestry College, Agricultural University of Hebei, Baoding, China; ^2^Langfang Academy of Agriculture and Forestry Sciences, Langfang, China; ^3^Hebei Key Laboratory for Tree Genetic Resources and Forest Protection, Baoding, China

**Keywords:** transgenic poplar, insect resistance gene, toxin protein expression, multigene transformation, biosafety

## Abstract

Poplar is one of the main afforestation tree species in China, and the use of a single, or only a few, clones with low genetic diversity in poplar plantations has led to increasing problems with insect pests. The use of genetic engineering to cultivate insect-resistant poplar varieties has become a hot topic. Over the past 20 years, there have been remarkable achievements in this area. To date, nearly 22 insect-resistant poplar varieties have been created and approved for small-scale field testing, environmental release, or pilot-scale production. Here, we comprehensively review the development of insect-resistant genetically modified (GM) poplars in China. This review mostly addresses issues surrounding the regulation and commercialization of *Bt* poplar in China, the various insecticidal genes used, the effects of transgenic poplars on insects, toxic protein expression, multigene transformation, the stability of insect resistance, and biosafety. The efficacy of GM poplars for pest control differed among different transgenic poplar clones, larval instars, and insect species. The Bt protein analysis revealed that the expression level of Cry3A was significantly higher than that of Cry1Ac. Temporal and spatial studies of Bt protein showed that its expression varied with the developmental stage and tissue. The inheritance and expression of the exogenous gene were reviewed in transgenic hybrid poplar progeny lines and grafted sections. Biosafety issues, in terms of transgene stability and the effects on soil microorganisms, natural enemies of insects, and arthropod communities are also discussed.

## Introduction

Since the first report on transformation of *Bt* gene into *Populus nigra* was published in 1991, transgenic approaches have been widely used in breeding trees for insect resistance and other environmental stress tolerance in China. China is the only country worldwide with longstanding significant commercial Bt poplar plantations, the area has increased to 450 hm^2^ since two Bt transgenic poplars were commercialized in 2001 (Lu and Hu, 2011). This means that the greatest experience of using GM poplar resides in China although studies of various species of insect-resistant plants are ongoing worldwide. We hope that this review will help develop the industry in other countries.

Genetic engineering allows rapid insertion of exogenous insect resistance genes into the plant genome and their expression in the plant. The poplar is a model tree species used in research on woody plant molecular biology, and genetic engineering of poplar has developed rapidly. In the time since the successful transformation of the poplar clone NC-5339 (*Populus alba* × *P. grandidentata*) with a synthase gene conferring resistance to the herbicide glyphosate in 1987 (Fillatti et al., 1987), additional transformation protocols yielding genetically modified (GM) poplars, including *Agrobacterium*-mediated and biolistic transformation, have been developed. A proteinase-inhibitor gene from potato was used early to confer insect resistance on poplars (McNabb, 1987). An insect toxin-encoding gene (*Bt*) of *Bacillus thuringiensis*, a common soil bacterium, became one of the most widely used genes and was successfully transferred into poplar in 1991 (McCown et al., 1991; Wu and Fan, 1991).

To date, insecticidal genes have been obtained from plants, animals, and microorganisms. Genes encoding proteinase inhibitors, phytolectin, amylase inhibitors, and chitinase, are of plant origin, being part of the natural defenses developed by plants to counter insect attack. Animal sources of insect resistance genes include wasps, spiders, scorpions, and mammals. *B. thuringiensis* (a soil bacterium) is a microbial source of insecticidal toxins. The effectiveness of insect resistance genes varies. Overall, genes from plants and animals tend to not confer prospective effects on poplars: some have hardly any effect (Confalonieri et al., 1998) and others produce low levels of insect resistance (Zhuge et al., 2003; Zhao et al., 2005). However, some have been associated with high-level pest mortality sustained over long periods of time (Leplé et al., 1995; Wu et al., 2000; Delledonne et al., 2001). The *Bt* genes have been the most studied and are the best understood. These genes have been extensively modified via removal of AT-rich regions and addition of tissue-specific promoters. Bt toxin is more toxic than other insecticidal toxins at the same levels (Schuler et al., 1998). The *Bt* gene family constitutes a large reservoir of genes encoding insecticidal proteins. Also, the “CryI and CryIII parasporal crystal proteins” have been extensively studied, targeting Lepidoptera and Coleoptera larvae, respectively (Tian et al., 1993; Bradley et al., 1995; Génissel et al., 2003; Klocko et al., 2013).

The major insect pest species currently causing economic loss and ecological problems in poplar in China are trunk borers and defoliators (insects of the Lepidoptera and Coleoptera). Some species reproduce several times a year. Coleopteran pests are often borers and leaf beetles such as *Anoplophora glabripennis* Motschulsky, *Apriona germari* Hope, and *Plagiodera versicolora* Laicharting. Lepidopteran pests include *Hyphantria cunea* Drury, *Lymantria dispar* Linnaeus, *Apocheima cinerarius* Ershoff, *Malacosoma neustria* Motschulsky, and moth species of the Limacodidae and Notodontidae. Therefore, research on *CryI* and *CryIII* and their transformation into poplar has been funded by the National High Technology Research and Development Program of China (“Program 863”). Gene modification and recombination have yielded efficiently expressed genes. Poplars expressing the CryI or CryIII *d*-endotoxin exhibit high-level resistance to Lepidoptera and Coleoptera pests. Insect lethality is high (up to 100%); the toxins also inhibit larval weight gain, retard development, and reduce insect foraging (Wang et al., 1996; Wang G.Y. et al., 2012a; Rao et al., 2000; Zhang et al., 2015).

## The Management and Commercialization of Insect-Resistant Poplars in China

Research on GM poplars in China commenced in the early 1990s; the first milestone was transformation of the *Bt* gene into *P. nigra* (Wu and Fan, 1991). Research efforts in this area continued focusing on white and black poplars and various hybrids (Zhang B. Y. et al., 2005; Hu et al., 2010). After two decades of study, there have been remarkable achievements in the fields of insecticidal gene transfer, toxin expression and transportation, multigene transformation, insect resistance sustainability and stability, and biosafety. The State Forest Administration (SFA) has established several regulatory frameworks for forest genetic engineering; all research on insect-resistant trees must follow certain procedures in terms of project application and approval, small-scale field testing, environmental release, pilot-scale production, and commercialization, each phase must be evaluated by experts (Lu and Hu, 2011). **Table 1** summarizes the approval process for insect-resistant poplars in China. By 2002, only two poplar clones, *P. nigra* carrying the *Cry1A* Bt-toxin gene and a hybrid white poplar (clone 741) with a fusion of the *Cry1Ac* and *API* genes (the latter encoding the arrowhead proteinase inhibitor from *Sagittaria sagittifolia*) had been approved for commercial production (Lu and Hu, 2006). Presently, nearly 22 insect-resistant poplar varieties have been created and approved for small-scale field testing, environmental release, or pilot-scale production. The ecological safety of transgenic plants has become a hot topic and a major obstacle to the use of transgenic plants. Regulatory issues related to transgenic plants concentrate on health, safety, and environmental risks, and especially on the problems associated with (direct or indirect) human or animal consumption of GM trees and their by-products. However, the results are inconclusive. Poplar is a perennial tree with a long growth cycle. It will be necessary to perform a long-term ecological risk assessment before beginning field trials and commercial production of poplars. Therefore, the Chinese government has adopted a cautious attitude; although many other insect-resistant poplar varieties have been obtained following development of the two above-mentioned varieties, no new commercial trees have been approved.

**Table 1:** The administrative approval process of insect-resistant poplars in China.

Gene	Poplar	Small-scale field testing	Environmental release and pilot-scale production	Commercialization
*Cry1Ac + API*	Poplar 741	1996–1998	1999–2001	2002–2007
*Cry1A*	*Populus nigra*	1996–1998	1999–2001	2002–
*Cry1A*	*Populus euramericana* cv. “Robusta 94”	2006–2009	2012–2015	
*Cry3A + OC-I + Vgb + SacB + JERF36*	*P. euramericana* “Guariento”	2005–2008	2009–2013	
*Cry3A*	*Populus alba* × *P. hopeiensis*	2005–2008		
*Cry1A*	*Populus deltoides* × *P. simonii*	2005–2008		
*Cry1A(b) + spider insecticidal peptide gene*	*Populus simonii* × *P. nigra*	2006–2010		
*Cry3A*	Poplar 741	2006–2009	2011–2014	
*Cry3Aa8*	*Populus euramencana* cv. “Nanlin 895”	2009–2012		
*Cry1Ac + API*	*Populus tomentosa* Carr. Clone BL73	2011–2014		
*Cry3A + Cry1Ac + API*	Poplar 741	2011–2014		
*Cry1Ac + API*	*Populus euramericana* cv. “74/76”	2011–2014	2016–2018	
*Cry3A + Cry1Ac + BADH*	*Populus euramericana* cv. “74/76”	2016–2018		
*Cry3A + Cry1Ac + NTHK1*	*Populus euramericana* cv. “74/76”	2016–2018		


## Efficacy of GM Poplars in Pest Control

Irrespective of the level of insecticidal proteins in transgenic plant tissue, insect bioassays are the most sensitive method of evaluation. A great deal of work may be summed up as follows.

### Tolerances Differ Among Larval Instars

Genetic modification of poplar trees affects pest mortality, feeding, growth, and development. However, the various larval instars of the Lepidoptera or Coleoptera differ in their tolerance to toxin. For example, when *P. versicolora* adults and larvae fed on leaves of eight poplar clones expressing different levels of the Cry3A *d*-endotoxin, the mortality of the 1st, 2nd, and 3rd instar larvae attained 100% after 1–2, 2–3, and 3–4 d, respectively, whereas adult mortality was only 0–15% after 1 d, attained a maximum of 95%, and in some clones was no more than 19% after 4 d (Wang G.Y. et al., 2012a; Wang et al., 2012b). When *C. anachoreta* larvae (instars 1–4) fed on leaves expressing Cry1Ac, lethal effects on older larvae (3rd and 4th instars) were not marked (mortality 22.22%), whereas the 1st and 2nd instars were greatly affected (mortality 100%); lethality declined with development. *H. cunea* larvae (instars 1–6) could reach 100% mortality, but there were differences among different instars, the 1st instar larvae died after 3 d, while the 6th instar larvae died after 7 d (Zhang et al., 2015). The larval body-length and head width differed among different larval instars. The mortality rates of different instar larvae vary according to the growth and development of the insects. In general, the later/higher the instar larval stage, the less sensitive is the instar to the toxin, and the longer the larval survival time (Gao et al., 2002; Guo et al., 2004). A GM clone highly toxic to 1st-instar larvae may not necessarily exert the same effect on older larvae. Therefore, it is not enough to evaluate a new insect-resistant plant clone using 1st instars alone; all other instars and adults (such as leaf beetles) must be evaluated also.

### Insects Differ in Terms of Toxin Sensitivity

Different insect species, even those in the same class and order, differ in terms of sensitivity to insecticidal proteins. Four Lepidoptera species were fed on *Bt* + *API-*transgenic poplar leaves; the toxin-sensitivities differed markedly. *Micromelalopha troglodyta* was the most sensitive, followed by *H. cunea*, *Clostera anachoreta*, and *L. dispar* (Gao et al., 2004a). The average rate of lethal effects of the 741 transgenic poplar clones pB29 and pB11 on *C. anachoreta* 1st-instar larvae was 75–95%, *H. cunea* was lethal in >95% of cases in most years (Ren et al., 2017). The poplar 741 clone CC84 expressing the *Cry3A* gene modified the behavior of Coleopteran insects. Feeding tests were conducted on *P. versicolora* and *A. germari*. Larvae of instars 1–3 of *P. versicolora* all died after 4 d of feeding (Wang G.Y. et al., 2012a), but no more than 50% of *A. germari* larvae died (Zhen et al., 2007; Niu et al., 2011b). This indicated that leaf beetles were more toxin-sensitive than long-horned beetles. Different insects, even those belonging to the same class and order, appear to have different enzyme systems according to variations in body size. Therefore, they showed differential sensitivity and tolerance to the toxic proteins.

### Transgenic Clones Differ in Insect Resistance

Different transgenic poplar clones differ in insect resistance. Three levels are usually recognized: high-level resistance, moderate resistance, and low-level resistance. High-level resistance is associated with major mortality (mortality > 80%) of larvae of all instars, regardless of developmental stage. Moderate resistance is characterized by lower mortality but consistent killing of larvae of all instars. Low-level resistance is associated with moderate or low-level mortality of the first 1–2 instars, but much lower mortality (mortality < 40%) and a longer latency to death of older larvae (Gao et al., 2004b; Zhang et al., 2015). The toxicities of 28 1-year-old greenhouse-grown clones of GM (*Cry1Ac* + *API*) triploid *P. tomentosa* clones were assessed. Insects (*L. dispar* Linnaeus and *C. anachoreta* Fabricius) fed on fresh leaves. These clones varied in terms of insect resistance: 11 subclones had a mortality > 80%, 7 had a mortality of 60–80%, 10 had a mortality < 50%, and some produced no toxic effects (Yang et al., 2006a). The bioassays were repeated 2 and 6 years after the clones were planted in a protected seedling nursery; the clones still varied in terms of insect resistance, which correlated closely with the data from 1-year-old seedlings (Yuan et al., 2007; Li et al., 2009). In a study including eight *P. euramericana* Neva clones expressing *Cry1Ac* + *API*, five clones killed all stage 1–4 instar larvae of *C. anachoreta*, and the other three clones killed all 1st–2nd instar larvae, but only 22.22% of instar 3rd and 4th larvae (Zhang et al., 2015).

Numerous factors can cause differences in the expression of the same transformation event. Molecular analyses of hybrid aspen revealed that transgene inactivation was always a consequence of transgene repeats (Kumar and Fladung, 2001). T-DNA repeats (the copy number of the exogenous gene in the host chromosome) influenced transgene expression differentially in different transgenic lines (Fladung and Kumar, 2002). The insert location and gene sequences around the integration site of the exogenous gene could also affect transgene expression (Dong et al., 2015).

Many studies have focused on the selection and utilization of transgenic clones with high resistance. Although this approach can control insect pests effectively, the genetic diversity and stability of the forest will be reduced as a result of using a single high insect-resistant clone (Ren et al., 2018). The recommended insect resistance management strategy involves use of a high dose and refuge strategy to slow the development of resistance to *Bt* plants in the target insect. However, designing mixed afforestation strategies using GM clones with high and medium insect resistance is more conducive to maintaining variety and stability and preventing insect tolerance (Andow and Zwahlen, 2006; Hu et al., 2010; Ren et al., 2018).

## Multigene Transformation

Most early work featured transformation with single toxin-encoding genes. As reports on the emergence of pest-tolerance and the low toxicities of certain proteins increased in number, multigene transformation (two or more toxin-encoding genes), and combinations of the *Bt* gene with other genes, have become increasingly popular in efforts to create cumulative insecticidal effects by expressing genes differing in terms of mechanism of action or specific binding site. The principal techniques are bivalent or multivalent vector construction, co-transformation, double transformation, use of a gene gun, and hybrid formation. **Table 2** shows multigene transformation of poplar varieties, and the insects used for testing, in China.

**Table 2:** Multigene transformation of poplar varieties, and the insects used for testing, in China.

Gene	Poplar variety	Tested insects	Reference
*Cry1Ac + API*	Poplar 741	*Lymantria dispar* Linnaeus	Zheng et al., 2000
		*Clostera anachoreta* Fabricius	Tian et al., 2000;Yang et al., 2005
		*Micromelalopha troglodyta* Graeser	Gao et al., 2004a
		*Hyphantria cunea* Drury	Gao et al., 2004a,b
	*Populus tomentosa*	*Clostera anachoreta* Fabricius	Yang et al., 2006a
		*Lymantria dispar* Linnaeus	
	*P. euramericana* cv. “74/76”	*Clostera anachoreta* Fabricius	Yang et al., 2012;Zhang et al., 2015


		*Hyphantria cunea* Drury	
	*Populus tomentosa* clone 85	*Lymantria dispar* Linnaeus	Li et al., 2007a
	*Populus alba* × *P. glandulosa* cv. “84K”	*Clostera anachoreta* Fabricius	Zhang B. Y. et al., 2005
*Cry1Ac + CpTI*	*P. euramericana* cv. “Nan-lin895”	*Micromelalopha troglodyta* Graeser	Zhuge et al., 2006;Zhang et al., 2012a
		*Clostera anachoreta* Fabricius	Zhang et al., 2012b
*Cry1A(b) + s*pider insecticidal peptide gene	*P. davidiana* Dode × *P. bollena* Lauche		Zuo et al., 2009
	*P. euramericana* 108	*Lymantria dispar* Linnaeus	Zou et al., 2010
	*Populus simonii × P. nigra*	*Lymantria dispar* Linnaeus	Jiang et al., 2004;Lin et al., 2006
		*Clostera anachoreta* Fabricius	Fan et al., 2006
		*Clostera anastomosis* linnaeus	Zhao et al., 2010
*Cry3A + OC-I*	*Populus alba* × *P. glandulosa* cv. “84K”	*Anoplophora glabripennis*	Zhang B. Y. et al., 2005
*Cry3A + OC-I + SacB + vgb + JERF36*	*P. euramericana* “Guariento”	*Anoplophora glabripennis*	Wang et al., 2007
*Cry1Ac + Cry3Aa + API*	Poplar 741	*Hyphantria cunea* Drury	Wang G.Y. et al., 2012a; Wang et al., 2012b
		*Plagiodera versicolora* Laicharting	
*Cry1Ac + Cry3A*	*P. deltoides* cv. “Juba”	*Hyphantria cunea* Drury	Dong et al., 2015
		*Plagiodera versicolora* Laicharting	
*Cry1Ac + Cry3A + NTHK1*	*P. euramericana* “Neva”	*Hyphantria cunea* Drury	Liu et al., 2016
		*Plagiodera versicolora* Laicharting	
*Cry1Ac + Cry3A + BADH*	*P. euramericana* “Neva”	*Hyphantria cunea* Drury	Yang et al., 2016
		*Plagiodera versicolora* Laicharting	


### *Bt* Associated With Proteinase Inhibitor-Encoding Genes

Proteinase inhibitors, which are natural insecticidal agents and one of the most abundant types of protein in nature, are found mainly in the storage organs of plants, especially seeds and bulbs (Liu and Xue, 2000). These low-molecular weight peptides/proteins interfere with insect digestion (Abe and Arai, 1991; Joanitti et al., 2006). Many relevant genes have been cloned and the effects of the gene products have been studied in insects in terms of poplar insect resistance (Leplé et al., 1995; Hao et al., 1999; Lin et al., 2002; Zhang et al., 2002; Confalonieri et al., 2003; Zhuge et al., 2003). In addition, combinations of *Bt* with proteinase inhibitor genes have been used to create pest-resistant poplar. *Cry1Ac* + *API* were together transferred into *P. tomentosa* Carr., *P. euramericana* cv. “74/76” poplar 84K (*P. alba* × *P. glandulosa* cv. “84K”), and poplar 741 (*P. alba* L. × *P. davidiana* Dode + *P. simonii* Carr. × *P. tomentosa* Carr.) (Tian et al., 2000; Zheng et al., 2000; Li et al., 2007a,b; Yang et al., 2012). *Cry1Ac* + *CpTI* (the latter gene encoding the cowpea trypsin inhibitor) were expressed in *P. euramericana* cv. “Nanlin 895” via co-transformation (Zhuge et al., 2006). *Cry3A* + *OC-I* (the latter gene encoding the rice cysteine proteinase inhibitor oryzacystatin-I) were transferred into *P. alba* × *P*. *glandulosa* via *Agrobacterium*-mediated transformation (Zhang B. Y. et al., 2005). All such GM clones were more toxic to young larvae and adults of target insects than poplar clones carrying the single genes. However, *Bt*-targeted insects were more sensitive, indicating that *Bt* played the major role (Li et al., 2000; Zhuge et al., 2003; Yang et al., 2006a).

### *Bt* Associated With a Spider Gene Encoding an Insecticidal Peptide

It is well-known that spider toxin can rapidly paralyze insects and mammals (Bloomquist et al., 1996). A spider insecticidal peptide purified from *Atrax robustus* Simon (Araneae: Hexathelidae) venom at Deakin University (Australia) contains 37 amino acids and kills many agricultural pests without harming mammals (Jiang et al., 1995). The gene encoding the toxin was artificially synthesized in the College of Life Sciences, Beijing University, in co-operation with Deakin University, preserving the amino acid sequence, but using plant-preferred codons. Cotton expressing the gene was toxic to *Helicoverpa armigera* and the growth of surviving insects was remarkably retarded (Jiang et al., 1996).

Work on transformation of the fused spider insecticidal gene and the C-terminal region of the *Cry1A*(*b*) gene into poplar became a priority of the Northeast Forestry University of China. *Populus simonii × P. nigra*, *P. davidiana × P. bolleana*, and *P. euramericana* 108 were transformed after 2000 (Jiang et al., 2004; Lin et al., 2006; Zuo et al., 2009; Zou et al., 2010). Leaves of *P. simonii* with both genes were fed to larvae of *C. anachoreta* and *L. dispar*. The growth and development of larvae were significantly affected; ecdysis was suppressed, the pupae were deformed, pupation was reduced, and pupal weight fell. Transmission electron microscopy of larval midgut showed that the larvae fed poorly and the midgut was deformed (pathological changes were evident in the columnar and goblet cells); toxicity increased over time (Fan et al., 2006; Cao et al., 2010; Zhao et al., 2010).

The insecticidal mechanisms of spider peptide and Bt protein are different. Theoretically, the combination of these two genes should improve insect resistance. There has been no report on the transformation of a single spider gene in poplar. Tobacco possessing a single spider peptide was toxic to *H. armigera*, with a mortality rate of 30–45% (Jiang et al., 1996). This rate is relatively low compared with that for Bt toxin. Therefore, the insect resistance specificity and environmental safety of the *Bt* and spider peptide combination requires further in-depth study.

### The Use of Two *Bt* Genes to Expand Insect Resistance

The *Bt* family contains many variants, and different toxins kill different insects (Tang et al., 2004). For example, the Bt toxins most commonly used, Cry1A and Cry3A, kill only Lepidoptera and Coleoptera, respectively. Therefore, combinations of Bt endotoxins in the same plants expand the insect resistance spectra, as validated in many studies (Meenakshi et al., 2011; Wang G.Y. et al., 2012a; Jiang et al., 2016). In China, two poplar varieties containing both *Cry1Ac* and *Cry3A* have been obtained. One is the hybrid poplar 741, another is the poplar Juba (*P*. *deltoides* cv. “Juba”). Ten hybrid poplar 741 clones with two insect resistance genes (*Cry1Ac + API*) were first created in 2000 (Tian et al., 2000), and bioassays of four clones (pB29, pB17, pB12, and pB11) used as food for *H. cunea* and *L. dispar* revealed obvious insecticidal effects (Gao et al., 2004b). In 2012, *Cry3A* was transferred into pB29 via *Agrobacterium*-mediated double transformation (Wang et al., 2012b). Compared with poplars expressing *Cry3A* or *Cry1Ac* alone, poplars with *Cry1Ac* + *Cry3A + API* were toxic to both Lepidoptera and Coleoptera (Wang G.Y. et al., 2012a; Wang et al., 2012b). Dong et al. (2015) constructed a plant expression vector containing both *Cry1Ac* and *Cry3A*, the processes involved a simple, rapid, and efficient genetic transformation technique. The transformation of poplar Juba showed that the genome obtained the two *Bt* genes simultaneously.

Apparently, the combination of two or more *Bt* genes with different insecticidal specificities can expand the insect resistance spectra. There are relatively few poplars with such characters and more research is needed to understand the potential associated benefits and problems.

### *Bt* Associated With Genes That Do Not Induce Insect Resistance

Genes that improve plant stress tolerance, such as drought- and cold-tolerance genes, salinity- and alkalinity-tolerance genes, and other stress-related genes, were cloned earlier and transferred to poplars in China (Yang et al., 2001; Fan et al., 2002; Sun et al., 2002; Zou et al., 2004). The search for new stress-tolerance genes (e.g., *eIF1A*, *DREB1C*, *GmNHX1*, and *OsNHXI*), and modification of the available genes to further improve poplar traits, are ongoing. The aim is to increase tolerance to both biotic and abiotic stresses (Li J. et al., 2010; Huang and Tian, 2011; Sun et al., 2013; Ji, 2015; Zhu and Wang, 2015). Transformation of *Bt*, together with other insect resistance genes, into such plants to confer multiple useful traits is also being studied.

Five cloned genes, a regulatory gene (*JERF*36), a levansucrase-encoding gene (*SacB*), the gene encoding the *Vitreoscilla* hemoglobin (*vgb*), and the “binary coleopterous insect resistance” gene (*Cry3A* + *OC-I*) were co-transferred into *P. euramericana* “Guariento” using a gene gun. Twenty-five kanamycin-resistant plants were obtained, of which seven contained all five genes as revealed by polymerase chain reaction (PCR) and Southern hybridization (Wang et al., 2007). These plants grew well on coastal saline soil in Dagang, Tianjin. Subsequent research showed that the plants were highly tolerant to drought, waterlogging, and salinity (Li H. et al., 2010; Su et al., 2011; Li et al., 2015).

*NTHK1* and betaine aldehyde dehydrogenase gene (*BADH*) are two salt-tolerance-related genes; *NTHK1* is an ethylene receptor gene from tobacco, and is induced by mechanical injury, NaCl, and PEG (Cao et al., 2006). The protein encoded by the *BADH* catalyzes the synthesis of glycinebetaine, an important quaternary ammonium compound produced in response to salt and other osmotic stressors by many organisms (Jia et al., 2002). Multigene plant transformation vectors carrying *Cry1Ac* + *BADH*, *Cry1Ac* + *NTHK1*, *Cry1Ac* + *Cry3A* + *NTHK1* (Du et al., 2014; Liu et al., 2014), and *Cry1Ac* + *Cry3A + BADH* (Yang et al., 2012, 2016; Liu et al., 2016) were created at the Hebei Key Laboratory for Tree Genetic Resources and Forest Protection, Hebei province. The genes were successfully transformed into tobacco and various poplars; regenerated plants exhibited both pest-resistance and an increase in salt tolerance compared with controls (Ren et al., 2015).

## Expression and Transport of Bt Proteins in Poplars

### Difference in Expression Levels of Bt Proteins

Measurements of Bt protein levels and the results of insect feeding tests revealed a close positive relationship between protein expression level and pest-resistance; clones expressing high Bt levels exhibited high-level insect resistance (Tian et al., 2000; Dong et al., 2015; Liu et al., 2016). To date, *Cry1Ac* and *Cry3A* of the *Bt* family have been most widely used for genetic transformation of insect-resistant poplars in China. Enzyme-linked immunosorbent assay (ELISA) analysis revealed significant differences in Bt protein levels; that of Cry3A was significantly higher than that of Cry1Ac, independent of the transformation method used (individual transfer of *Cry1Ac* and *Cry3A*) (Tian et al., 2000; Yang et al., 2006a; Wang et al., 2008; Niu et al., 2011b), transformation of *Cry3A* into a plant already expressing *Cry1Ac* (Wang G.Y. et al., 2012a), or transfer of *Cry1Ac* and *Cry3A* together (two *Bt* genes were constructed in one vector and then inserted into the poplar genome) (Dong et al., 2015; Zhao et al., 2016; Liu et al., 2016). The toxic protein expression levels of the two *Bt* genes differed significantly between the acquired transgenic clones. The expression level of Cry3A (2.24–13.30 μg g^-1^ FW) was typically 1,000-fold greater than that of Cry1Ac (16.44–60.32 ng g^-1^ FW) (Wang G.Y. et al., 2012a). In most of these studies, CaMV35S promoter was employed. Dong et al. (2015) tried to find the differences between different promoters and vectors, in their transformation of poplar Juba, two Bt genes (*Cry1Ac* and *Cry3A*) on vector p71A68Y71 were separately driven by promoters CAMV35S and CoYMV, and a matrix attachment region (MAR) sequence was added to both sides; in contrast, two Bt genes on vector p05A68A71 were driven by the promoter CAMV35S without a MAR sequence structure. The result also showed that Cry3A toxic protein content was much higher than the Cry1Ac toxic protein content in all regenerated lines.

The difference in Bt expression levels may be related to the sequence and direction of exogenous vector genes, the exogenous genes species, or the promoter type (Ren et al., 2015). When two genes were constructed in the same vector, the insertion was adjacent. Because of the high homology of the two genes, interference was possible (because one gene inhibited the other). In addition, the arrangement order of the targeted genes may also affect gene expression. Studies on tobacco showed that, when two *Bt* genes were simultaneously constructed in one transformation vector, the *Bt* gene close to the left boundary of T-DNA was efficiently expressed, regardless of whether it was *Cry3A* or *Cry1Ac*, whereas the gene close to the right boundary was inhibited (Dong et al., 2015). This finding requires further research and validation.

### Temporal and Spatial Dynamics of Bt Protein Expression

Temporal and spatial studies of Bt protein expression showed that expression varied by the developmental stage and the tissue. The Cry3A protein levels of twig xylem, and the roots of a 2-year-old poplar 741 clone, increased consistently, whereas the leaf level first decreased and then increased over the growing season. In terms of tree crown layers, the Cry3A protein level in xylem increased from the upper to the lower crown, but the leaf pattern was the opposite (Niu et al., 2011a). The temporal/spatial dynamics of Cry1Ac protein expression were also monitored in a plantation of 6- to 8-year-old trees of a transgenic insect-resistant poplar. Cry1Ac protein content changed in a consistent manner, initially increasing and then decreasing over the growing season (6–10 months) of each year, peaking in August, and then decreasing. The levels of Cry1Ac protein were, in rank order: root system > leaves of short branches > leaves of long branches (Zhang et al., 2016). The expression regularity of Bt protein is closely related to the tree growth pattern of *Bt* poplar, in fast growing season and metabolism tissues, Bt protein expression is high accordingly.

### Transport of Bt Protein in Transgenic Poplars and Heredity of Exogenous Genes in Transgenic Hybrid Progeny Lines

Wang and Yang (2010) and Wang L.R. et al. (2012) grafted non-transgenic and transgenic poplar 741 (with the *Cry1Ac* gene) samples as both scions and stocks, and Cry1Ac protein transportation studied using ELISA. The protein was detected in non-transgenic tissue (leaf, phloem, xylem, and pith), being especially high in phloem, indicating that the protein moved from the stock to the scion of grafted plants, principally through the phloem. The protein moved from the roots (stock) to upper parts of the plants (scions), and vice versa. When leaves of grafted branches of non-transgenic 741 poplars were fed to *C*. *anachoreta* larvae, insect development was delayed. After 8 years of field growth, the transport and accumulation of the Bt protein (in terms of sites of occurrence, levels, and direction of movement) in grafted adult poplars were studied once more. Although some changes were evident, most toxin was transported and accumulated as found previously (Chen et al., 2016).

In the above study on poplar, ELISA analysis showed that accumulation of Bt protein was highest in phloem tissues, indicating that Bt protein was mainly translocated within the phloem across the graft union (rootstock, scion, and interstock) to the leaves. Reverse transcription-PCR (RT-PCR) showed that mRNA of *Bt* gene was not detected in the branch and leaf of non-transgenic poplar 741 no matter its material was used as scion or stock, which suggested that mRNA of *Bt* gene was not transported between the stock and scion (Wang and Yang, 2010).

## Biosafety Assessment

Genetic modification technology should provide substantial economic and long-term environmental benefits. Over the past 30 years, various GM trees with modified characteristics have been created. The wide application of transgenic technology to tree genetics and breeding has greatly accelerated progress (Häggman et al., 2013). Potential risks such as unwanted gene flow and pleiotropic effects of transferred genes attract increasing public attention (Andow and Zwahlen, 2006; Hoenicka and Fladung, 2006). The safety issues include the effects of transgenic poplars on soil microbes, the natural insect enemies of poplar pests, non-target insects, and arthropods as well as transgene stability and the possible impacts of vertical and horizontal gene transfer.

### Field Testing and Stability of Transgenes

Culture and regeneration of transgenic plantlets is performed in the laboratory. Transgenic plants are rapidly micropropagated *in vitro* and seedlings with roots are then planted in experimental fields being subjected to adaptive exercises mimicking the external environment. Many reports on annual crops have shown that transgene expression is less stable than originally thought (Meyer, 1995). Trees are perennials with long life-cycles, raising concerns about the stability of exogenous genes and the long-term field efficacy of transgenic tree growth (Hawkins et al., 2003).

The inheritance and expression of the exogenous Bt gene/protein was studied in transgenic hybrid poplar progeny lines. Hybridization was implemented using non-transgenic poplar 84K as the male parent trees, and transgenic poplar 741 lines showing different insect-resistant ability [high insect resistance (pB11, pB29), moderate insect resistance (pB1, pB17), and no insect resistance (pB6)] were used as the female parent trees. The insect resistance of the hybrid progeny plants was nearly identical to that of the parent plants (Ren et al., 2017). This study indicated that using transgenic poplars as parent plants for sexual hybridization could transfer exogenous genes to progeny, and lead to the cultivation of new insect-resistant species.

Polymerase chain reaction, Southern blotting, and ELISA showed that exogenous genes were stable for many years in field-planted GM poplars (Wang et al., 1996; Lin et al., 2002; Zhang et al., 2004; Yang et al., 2006a). Three poplar 741 (*Cry1Ac + API*) plantations exhibiting high-level insect resistance were exposed to *C. anachoreta*, *L. dispar*, *H. cunea*, and *M. troglodyta* over 4 successive years. Insect mortality fluctuated somewhat, but no regular decline was noted (Yang et al., 2005). Within the same year, different lines showed different degrees of resistances to the target pests, while differences within the same lines among years were also seen, which may be related to the climate, environment factors, and target pest status, among other factors (Ren et al., 2017). In the 8- and 10-year-old plantations, the diameter at breast height (DBH) of transgenic poplar 741 was also compared with that of non-transgenic poplar. DBH growth in non-transgenic poplar 741 did not differ significantly from that of transgenic lines. This indicates that the exogenous *Bt* gene had no influence on the growth of *Bt* poplar (Ren et al., 2017).

Compared with non-GM poplars, GM poplars protect their foliage from pest outbreaks in the wild. Transgenic poplar trees (*P. nigra*) expressing the *Cry1Ac* gene were evaluated in the field at the Manas Forest Station of the Xinjiang Uygur Autonomous Region during 1994–1997 and 1997–2001. The average proportion of severely damaged leaves on transgenic trees was 10% whereas leaf damage of control trees in nearby plantations attained 80–90%. The average number of pupae per m^2^ of soil at 20-cm depth in the test field declined from 18 to 8 pupae per m^2^ from 1994 to 1997, but increased in control plantations. In a field trial conducted in 2005 in Huairou, Beijing, Bt-expressing *P. nigra* exposed to *A. cinerarius* exhibited ≤20% foliage damage; the figure for control poplars was up to 90% (Hu et al., 2001, 2007).

The integrity and expression stability of exogenous genes in recipient plants are the primary issues in plant genetic engineering research, and are important factors in the application of genetic engineering technology. Under certain circumstances, after the exogenous gene is inserted into the plant, environmental factors may influence the expression stability of the exogenous gene (Brandle et al., 1995). In the process of gene transformation, changes can occur in the exogenous gene fragment inserted in the host chromosome, which may influence the growth and economic traits of plants. Data from the field plantation of transgenic poplar showed that the exogenous genes were stable in the transgenic trees, and the insect resistance of the transgenic lines did not show a downward trend over time.

### Survival and Escape of Agrobacterial Vectors

Genetic transformation is usually mediated via agrobacterial vectors (80% of all GM plants) (Wang and Fang, 1998). Such recombinants could escape into the soil if they survive in GM plants, rendering it possible that plasmids could move among soil microorganisms. When triploid *P. tomentosa* transformed with *Cry1Ac + API* was examined during subcultivation and after transplantation, residual agrobacteria were detected in 3 of 28 culture flasks of cultivars after 24 months of cultivation. The three strains were transplanted into pots for greenhouse cultivation. Agrobacteria were detected in the soil of one pot after 1 month. Thus, GM plants transformed using agrobacteria should be strictly checked before release, and strains carrying the recombinant agrobacteria must not be released (Yang et al., 2006b).

### Effects on Soil Microorganisms

There are two main routes by which proteins released from transgenic plants may enter the soil. One is via pollen or litter, and the other is via root exudates GM poplars were studied in the field in terms of residues, decomposition, and the influence thereof on soil microorganisms. Three groups of microbes (bacteria, fungi, and Actinomycetes) of *P. alba × P. glandulosa* trees transformed with *Cry3A* were investigated in the first and second years after transplantation. Analysis of variance (ANOVA) and multiple comparison analyses revealed no significant difference in the levels of soil microorganisms under most poplar lines at the same time points (Hou et al., 2009). In a field of 3-year-old poplars with the *CPTI* gene, no extraneous *CPTI* DNA was detected (Hu et al., 2004). The microbial compositions of soil at depths of 0–20 and 20–40 cm did not differ between the GM poplar and control plantations (Zhang Q. et al., 2005).

The toxin protein was found in the soil of 4-year-old transgenic poplar 741 (*Cry1Ac + API*) test fields, but the levels showed a descending trend, at each step in the rank order: root tissue > root surface soil > rhizosphere soil > surface soil. The Bt toxin protein distribution was not associated with microbial levels (Zhen et al., 2011). Studies of the microbial diversity of 5-year-old transgenic poplar 741 showed that transgenic poplars did not affect the physical and chemical properties of the soil or the soil microbial community structure. However, the microbial community structure was obviously affected by the location and season (Zuo et al., 2018).

The levels of the three groups of microbes in the soil of stands of 7-year-old transgenic *P. nigra* carrying the *Bt* gene did not differ from those of control stands (Hu et al., 2004). One study investigated a 10-year-old plantation of *P. euramericana* “Guariento,” land with five transgenic poplars, land with non-transgenic poplars, and land without any plants (NP). The transgenic poplar was found to have no significant adverse effects on the soil microorganism system. Non-transgenic poplars and transgenic poplars can both increase the metabolic activity of rhizosphere soil microbes compared with NP soil (Zhu et al., 2015).

Assessment of the impact of genetically engineered organisms on the rhizosphere microbe population in soil has become a hot topic. Numerous studies have been reported on the effect on soil microorganisms: most conclusions were positive, i.e., they had no obvious affects. The influence of transgenic trees and tree litter on microbial communities is dependent on the field site, season, and method used to assess the community. Due to the functional differences among different insect resistance genes, the impact of the expressed toxin protein on soil microorganisms may also differ. Rhizosphere microbes can perceive variations in plant root exudates (Liu et al., 2003). Future work needs to address the long-term effects of transgenic trees; these effects should not only be compared with those of non-transgenic counterpart, but also with other possible changes in the agroecosystem (Dunfield and Germida, 2004).

### Influence on Natural Enemies of Insects

To clarify whether transgenic poplars affect the levels of natural enemies of insects, laboratory feeding experiments were conducted; the ladybird *Harmonia axyridis* (Pallas) was fed in the laboratory on the aphid *Chaitophorus populeti* (Panzer) which had fed on leaves of poplar 741 (with *Cry1Ac + API*) (Yao et al., 2006) and *P. alba* × *P. glandulosa* (with *Cry3A + OC-I*) (Zhang et al., 2009). The results indicated that aphid consumption of transgenic plant food exerted no significant effect on the mortality, body mass, eclosion, sex ratio, or developmental times of the larval and pupal stages of *H. axyridis*. Studies showed that transgenic poplars with target insect resistance have no negative effect on the predatory ladybird. It is possible that the potential transfer of toxic proteins from the transgenic poplar via its aphid prey to the predator does not occur or, if it does, is not toxic to the predator. Further studies must be conducted to verify these results.

The population dynamics of the principal pests and their natural insect enemies in stands of transgenic poplars have also been studied. In *Bt*-transgenic *P. nigra* stands, the variety, number, and parasitic ratios of the natural enemies of insects were higher than those in non-transgenic plantations. Inoculation of insect pupae collected from transgenic, adjacent, and control plantations with the wasp *Chouioia cunea* Yang revealed no significant among-stand difference in wasp eclosion rate or number (Hu et al., 2007).

The target pests *L. dispar* and *Lymantria susinella* were well-controlled by the Cry1Ac + API proteins of transgenic poplars, and the non-target insect *Chaitophorus populialbae* was not affected; the population of this insect remained stable. In poplar stands exhibiting high- and medium-level insect resistance, the levels of *Vulgichneumon leucaniae* Uchida, a natural insect parasite, declined significantly; the parasite possibly moved away because of reduced prey levels. Compared with the control levels, those of the predators *Misumenops tricuspidatus* and *H. axyridis* Pallas fluctuated to some extent (Jiang et al., 2009).

It is clear that the insect-resistant poplar can cause the death of the target insects, which will likely have either a positive or negative influence on their natural enemies. The insect community forms a complex food chain. Studies of the interactions between transgenic poplar and the natural enemies of insects not only provide useful information about the ecological safety assessment but also suggest better methods for future biological control of insects, including target and non-target insects.

### Effects on Arthropod Communities

Arthropod communities are important components of tree ecosystems. Large time spans of continuous insect feeding in *Bt* poplar plantations have attracted investigation, to determine whether insects have developed resistance/tolerance to various controlling factors, and whether the total pest populations within arthropod communities have changed. Studies are typically conducted during the growing season. Plants were selected randomly according to the size of the experimental plantations. Each tree was divided into three layers (upper, middle, and lower), and each layer was divided into four directions: East, South, West, and North. The arthropod community distribution (vertical and horizontal) was surveyed in detail on the ground and in bark and branches. Species richness, dominance, evenness, diversity, and similarity were calculated and analyzed, using the Berge–Parker index, Shannon–Wiener index, evenness index, Simpson’s inverted index, and Bray–Curtis dissimilarity index. The differences between transgenic and non-transgenic poplars were also compared (Gao et al., 2003; Zhang et al., 2011; Guo et al., 2018; Zuo et al., 2018).

Experimental plots of 84K poplar were established in Beijing, China, in 2005, containing 84K poplar clones (BGA-5) with the *Cry3A* gene and non-transgenic 84K poplars as a control (CK). During the 3-year study (2006, 2007, and 2008), 4,956 arthropod individuals were observed in field trials, including 2,552 individuals on CK trees and 2,404 on BGA-5 trees. These arthropods belonged to 10 orders and 41 families, and included three functional guilds, e.g., phytophages, predators, and parasitoids. Arthropods of 37 families were found on CK; Lasiocampidae, Aegeriidae, Coreoidea, and Miridae were absent on CK trees, but were observed on BGA-5 trees. Although the families and individuals within functional arthropod groups observed in both populations were different, the dominant families in each guild were similar. The *Cry3A*-mediated reduction of the target pest (*P. versicolora*) was not associated with any effect on a non-target pest (*C. anachoreta*), and, generally, exhibited no significant negative effect on the poplar arthropod community (Zhang et al., 2011). These results suggest that planting *Bt* poplar generally had no significant negative effect on the poplar arthropod community.

A study in a transgenic poplar 741 (*Cry1Ac* + *API*) experimental forest (3-year-old trees) at a coastal forest farm (Tangshan, China) reported reductions in the numbers of defoliating insects, fewer dominant species, increased insect diversity, and evenness in terms of the insect pest sub-community. In the arthropod communities of transgenic poplar clones (pB1, pB3, pB11, pB17, and pB29), insect resistance was found to have a negative relationship with community, similar to that of control poplars (Gao et al., 2003). In the soil, surface, and shrub-grass layers, the arthropod sub-community structures of the high-insect resistance poplar 741 clone were near-normal within the soil layer. The other arthropod communities (surface and shrub-grass) exhibited higher diversity and uniformity indices and greater stability, but a low dominance concentration index (Gao et al., 2005).

Two-year-old *Bt* poplar 741 clones pB29 (*Cry1Ac + API*) and CC84 (*Cry3A*) were planted at the Ninghe nursery (Tianjin, China) in 2011. The arthropod communities in the experimental forest were determined over 4 consecutive years (2012–2015). A total of 21,662 insects from 12 orders were recorded. The arthropod community in transgenic poplar 741 trees was similar in structure and composition to that in control poplar 741 trees. The dominant insect species in all poplars was Hemiptera, followed by Lepidoptera, Homoptera, Araneida, and Diptera, whereas numbers of Mantodea, Neuroptera, Odonata, and Acarina were relatively low. The main poplar pests (primarily Lepidoptera) were significantly inhibited in the transgenic poplars; however, the number of Coleoptera pests was generally low, and the inhibitory effect was not clear (Zuo et al., 2018).

Experimental plots containing poplar 107 (*P. euramericana* “Neva”) with *Cry1Ac + API* were established in 2013 in the Luannan nursery (Tangshan, China). Four clones were investigated in 2015, using 2-year-old trees. In total, 6,818 insects belonging to 2 classes, 8 orders, 43 families, and 58 species were recorded. The dominant species on *Bt* poplar were in the Lepidoptera, Coleoptera, Hymenoptera, and Diptera families, with Lepidoptera species the most abundant. The number of herbivorous insects was significantly lower in transgenic poplar 107, whereas the number of sucking insects was significantly higher (Guo et al., 2018).

The characteristics and compositions of food webs in the arthropod communities were also studied. Compared with that of the non-transgenic poplar 741, the arthropod community of the GM poplar 741 exhibited more nutritional and species diversities, more net complexity, and more generalized information diversity, associated with a strong capacity to resist exogenous interference and to recover rapidly from disturbance (Yao et al., 2014). Many studies use the Shannon–Wiener diversity index to measure the amount of entropy or information in a system (Béla, 1995). However, this method was criticized for being sensitive to sample size and completeness, or for producing counter-intuitive community ordering in some cases (Butturi-Gomes et al., 2014). Guo (1988) made an improvement on the basis of Shannon diversity index and so-called generalized information diversity index, which can be decomposed into nutritional level diversity, species diversity, and network diversity (each has its calculation formula). These diversity indexes focus on the status and roles of species in food webs. Both simple and complex food webs have their fundamental characteristics, and these basic characteristics can reflect the internal changes in the food web structure.

No insect resistance was found after continuous long-term exposure during the period considered in China at present. One reason for this finding is that insect pests do not necessarily feed only on the transgenic trees but they may migrate to other plants diluting the exposure to Bt toxins. Another possible explanation is that the experimental plantations of *Bt* poplar were planted using a randomized block design and the area of each treatment was relatively small, i.e., the field trial of *Bt* poplar clone BGA-5 and the CK was designed in plots of 100 trees for each treatment (10 rows, 10 columns) with 2.0 m intervals between trees. In the *Bt* poplar 741 experimental forest, 25 strains were planted per plot, with three replicates; plants were spaced 2 m × 4 m apart. *Bt* poplar 107 (four transgenic clones) and the control poplar were planted with 30 strains per plot, with four replicates; plants were spaced 2 m × 5 m apart. These experimental designs reduced exposure of the target pests to Bt toxins. If large plantations of GM poplar are permitted in the future, it is likely that selection pressure will be much higher so that pests are more likely to evolve resistance.

Several strategies may be adopted to manage resistance. Establishing separate shelter or mixed shelter is a typical management strategy. Non-transgenic poplar plantations in or around GM trees can provide a sufficient number of pest-sensitive populations to dilute the resistance genes and delay insect resistance. Another approach is to plant poplar intercrops with crops such as cotton. Increasing the toxin expression levels of insecticidal genes and using multiple genes with different mechanisms can also delay the development of resistance. Using tissue-specific or time-specific insecticidal promoters, exogenous genes can be expressed in specific tissues or during specific tree developmental periods, such as during the insect hazard peak, and would be an ideal strategy for the temporal and spatial expression of Bt toxin. Fully assessing the risks of *Bt* poplars prior to commercialization poses great challenges, requiring multiple studies spanning numerous sites and successive generations of trees to evaluate ecological performance.

The stability of arthropod community characteristics in insect-resistant poplar plantations is an important consideration with respect to the safety of transgenic insect-resistant poplars. The arthropod community is a complex food web. Species diversity and quantity are fundamental parameters of the arthropod community; any variation therein will influence the entire community. Field tests are an obligatory early step toward future commercial deployment of GM trees. Monitoring should be part of the general stewardship and conditions for the release of a GM tree. A program offering stronger and more complete monitoring of insect resistance could provide a reliable basis for the implementation of the proposed strategy. Moreover, the safety of transgenic insect-resistant poplars must be assessed continuously. Further studies are required to obtain more detailed data for the biosafety evaluation of transgenic poplar.

### The Effect of an Insect-Resistant Poplar-Cotton Ecosystem on the Structure of the Arthropod Community

In the 22 years that have elapsed since insect-resistant cotton was first commercialized in China (James, 2008), cotton farmers have been afforded the obvious benefits of a reduced need for chemical pesticides, a cleaner environment, increased yield, and greater profit (Qiao, 2015). Poplar-cotton agro-ecosystems are common in China (Meng et al., 2004). According to the ISAAA 2016 report, 3.7 million ha of *Bt* cotton and 5.43 million ha of *Bt* poplar were planted in China in 2015 (James, 2015). As increasing areas of land become devoted to transgenic insect-resistant poplar and cotton, studies examining the effects of transgenic plants on target and non-target insects become increasingly important. The transgenic poplar-cotton ecosystem strongly inhibits insect pests, but has no impact on the structure of the arthropod community. The character index of the community indicated that the structure thereof was better than that of a control poplar-cotton ecosystem. In terms of the abundance of nutritional classes, the transgenic poplar-cotton ecosystem was also better than a non-transgenic ecosystem (Zhang et al., 2015a,b). This study further demonstrates the safety of transgenic plants, although the effects of insect-resistant poplar-cotton ecosystems on the arthropod community require comprehensive study, including continuous monitoring and tracking over the long term.

## Outlook

### Additional Insect Resistance Genes

*Bt* genes are the principal genes used to transform poplars. To date, >700 *Cry* gene sequences encoding crystal proteins have been identified (Palma et al., 2014); the encoded proteins kill Lepidoptera, Coleoptera, Diptera, and Hymenoptera in the field (Sanchis, 2011). Other anti-pest genes include those encoding plant protease inhibitors and plant agglutinins, non-*Bt* genes from bacteria (*Serratia entomophila*, *Pseudomonas entomophila*, and *Morganella morganii*), and genes from fungi [*Metarhizium anisopliae* (countering beetles or locusts), *Beauveria bassiana*, and *B. brongniartii*]. However, many insect pests are not susceptible to the products of these genes or are only poorly controlled thereby. Thus, further research is necessary to identify more efficient insect resistance genes.

A completely different approach for forest pests control is to use RNA interference (RNAi). RNAi is a biological process in which RNA molecules inhibit gene expression or translation, by neutralizing targeted mRNA molecules (Hannon, 2002; Kupferschmidt, 2013). The first step of using RNAi should be the selection of appropriate target genes, enzymes, or receptors which may cause abnormal growth, development, or reproductive inhibition of target pests. RNAi technology had been successfully applied in the study of various types of insects in recent years and many suitable target genes have emerged, such as chitin synthesis-related enzymes (chitin synthase and chitinase gene) (Arakane et al., 2005; Chen et al., 2008), hormones and receptors related to growth and development (epidermal hormones receptors EcR and juvenile hormones) (Huang et al., 2013), enzymes that modulate energy metabolism (proton metabolized V-ATPase and cytochrome P450 enzymes that metabolize toxic substances) (Tang et al., 2012; Kotwica-Rolinska et al., 2013), as well as neuromodulation pathways receptor and related enzymes (Agrawal et al., 2013). RNAi-mediated down-regulation of poplar had been studied and proved that RNAi is also functional in poplar (Mryer et al., 2004; Bi et al., 2015).

### Improving Multigene Transformation and Expression Systems

Highly effective multigene transformation systems for plants have been developed; vectors simultaneously express several target genes from a single plasmid (Chung et al., 2005). Many technical problems have been overcome, such as limitations in terms of multi-cloning and restriction enzyme sites, vector capacity, the need for dephosphorylation during construction, and end-filling of sticky ends with dNTPs. These steps are time-consuming, difficult, and tedious. Easy-to-use vector systems for multigene expression remain technically challenging. Conventional approaches for delivering foreign DNA include *Agrobacterium*-mediated and biolistics transformation, both of which result in the random integration of one or more copies of the DNA sequence (Baltes and Voytas, 2015). In recent years, genome editing technologies using sequence-specific nucleases have been developed as effective genetic engineering methods to target DNA at specific locations in plant genome (Fan et al., 2015). Genome editing technology can target to specific genomic sites by providing the means to modify genomes rapidly in a precise and predictable manner (Bortesi and Fischer, 2015). Furthermore, multigene interaction problems in transgenic plants require attention. Multigene transformation systems and their efficient expression in trees, such as the poplar, must be further explored and perfected.

### Biosafety Marker Genes and Marker-Free Poplar Transformation

Marker genes encoding antibiotic- or herbicide-resistance are often used to select transformed plant cells or tissues after exogenous gene transformation. Once the GM plant is obtained, the marker gene is both unnecessary and undesirable (Ye et al., 2012), complicating further transformation using the same selectable gene (Scutt et al., 2002). Therefore, selective marker gene elimination (marker knock-out) is of increasing interest. Although genomics has afforded a theoretical basis for the rapid and effective removal of marker genes, the process is not trivial (Ni, 2007). Selectable marker-free techniques are of great interest to those who work in plant biotechnology (Gu et al., 2014; Woo et al., 2015), but marker-free transformation to obtain pest-resistant poplars is in its infancy and more work is required.

### Creating New Poplar Varieties Using Gene Editing

Gene-editing, or genome editing developed in recent years is a type of genetic engineering in which DNA is inserted, deleted, labeled, modified, or replaced in the genome of a living organism (Maeder and Gersbach, 2016). To date, there are mainly four classes of sequence-specific nucleases for genome editing: meganucleases, zinc finger nucleases (ZFNs), transcription activator-like effector nucleases (TALENs), and the clustered regularly interspaced short palindromic repeat (CRISPR)/CRISPR-associated protein (Cas) (Gilles and Averof, 2014; Baltes and Voytas, 2015). They can be used to knock out genes or to introduce designed sequences into the genome with far greater efficiency than traditional genetic engineering strategies (Maeder and Gersbach, 2016).

The latest and most advanced technology is CRISPR–Cas9 genome editing technology which originates from type II CRISPR–Cas systems (Doudna and Charpentier, 2014). Since the introduction of CRISPR–Cas9, genome editing has become widely used in transformable plants for characterizing gene function and improving traits, mainly by inducing mutations through non-homologous end joining of double-stranded breaks generated by CRISPR–Cas9 (Yin et al., 2017). Two endogenous phytoene dehydrogenase (PDS) genes in *P. tomentosa* Carr., *PtoPDS* 1 and *PtoPDS* 2, had been knocked out simultaneously using the CRISPR–Cas9 technology, the result indicated the possibility of introducing mutations in two or more endogenous genes efficiently and obtaining multi-mutant strains of Populus using this system (Fan et al., 2015; Liu et al., 2015). Gene-editing techniques also render it possible to improve the pest-resistance of poplars by strengthening endogenous defenses. Highly expressed genes, or specific promoters, can be inserted into the poplar genome to precisely replace native genes or promoters. Mutant plants exhibiting specific high-level expression of insect resistance genes are thus created. Enhancing the expression of certain substances or increasing insect resistance by replacing promoters in poplar is only a scenario that requires further study and verification.

### Responsible Use of Anti-pest Genes

Lmitations in the insect resistance in transgenic plants have attracted the attention of researchers both in China and elsewhere (Bates et al., 2005; Christou et al., 2006; Wang et al., 2012b). Associated risks may be reduced using genetic strategies, refuge strategies, protection of genetic diversity, and barrier and field protection (Gao et al., 2003; Andow and Zwahlen, 2006). Many strategies have been applied to agricultural GM plants, while little work has examined forest trees. The strategy of using multiple genes with different characteristics to improve poplar insect resistance and expand the spectra has been proven effective in practice. The introduced gene must be expressed not only in the short term, but also in the long term. Insect resistance/tolerance has not yet been reported in poplar target species, and transgenic poplar lines showed no decrease in these traits after many years of field planting. The interaction between the transgenic plants and the main insect species is still insufficiently understood. Responsible pest management is a long process requiring continuous accurate monitoring.

## Author Contributions

MY proposed concept and edited the manuscript. GW and YD collected data and wrote the manuscript. XL, GY, and XY collected data and edited the manuscript.

## Conflict of Interest Statement

The authors declare that the research was conducted in the absence of any commercial or financial relationships that could be construed as a potential conflict of interest.
